# Deep learning for genomic insights into athletic performance in sports education

**DOI:** 10.3389/fgene.2026.1766127

**Published:** 2026-05-15

**Authors:** Bo Dong, Qing Lv

**Affiliations:** 1 Wuhan College, General Education Department, Wuhan, Hu Bei, China; 2 Capital University of Physical Education and Sports, Institute of Physical Education and Training, Beijing, China

**Keywords:** athletic performance prediction, constrained optimization refinement, deep learning, genomic factors, uncertainty aware prediction

## Abstract

**Introduction:**

This study proposes a deep learning based framework to investigate genomic factors influencing athletic performance in sports education. Traditional approaches often face challenges in modeling the high dimensionality and complex genotype phenotype relationships inherent in genomic data.

**Methods:**

To address these issues, the proposed framework integrates three core components: a formalized problem formulation, a Genomic Athletic Predictor, and a Constrained Optimization Refinement mechanism with uncertainty aware prediction. The method models the genomic feature space and athletic performance metrics under manifold informed constraints, while explicitly incorporating uncertainty quantification to enhance reliability. The Genomic Athletic Predictor consists of a Manifold Informed Constraint Encoder, an Agent Driven Genomic Planner, and an Uncertainty Guided Athletic Forecaster, enabling structured representation learning and robust performance prediction.

**Results and Discussion:**

Experimental evaluations conducted on two large scale cohort datasets demonstrate that the proposed framework consistently outperforms classical regression models, ensemble learning methods, and advanced neural network baselines. The model achieves superior results across multiple evaluation metrics, including Pearson correlation, RMSE, R^2^, and MAE, while maintaining moderate computational complexity. Ablation studies further confirm the complementary contributions of manifold constraints and uncertainty modeling in improving predictive stability and biological plausibility. These findings highlight the effectiveness of integrating deep learning, domain constraints, and uncertainty aware modeling for genomic based athletic performance prediction, offering practical implications for personalized training, talent identification, and data driven optimization in sports education.

## 1 Introduction

The study of athletic performance has long been a central focus in sports education, as understanding the determinants of physical excellence can significantly enhance training strategies, injury prevention, and talent identification Chen and Min (2025). Among these determinants, genomic insights have emerged as a promising avenue for uncovering the biological foundations of athletic ability Jianjun et al. (2025). Genomic data not only provide a deeper understanding of individual genetic predispositions affecting physical performance but also enable the design of personalized training regimens tailored to an athlete’s genetic profile More et al. (2026). Integrating genomic insights into sports education has the potential to transform athlete training and management, promoting a more scientific, data driven approach to performance optimization Spanakis et al. (2025). However, the complexity of genomic data and the challenges in interpreting its relationship with athletic traits necessitate advanced computational approaches. This has led to the evolution of methodologies from traditional symbolic AI approaches to modern deep learning techniques, each addressing specific limitations of prior methods Jia et al. (2025).

Initially, efforts to analyze genomic data in the context of athletic performance focused on structured approaches that utilized predefined rules and expert knowledge to model genetic influences on physical traits Li (2022). These methods provided interpretable insights by leveraging domain expertise, yet they were limited by their reliance on manually crafted rules, which restricted scalability and adaptability Li et al. (2025). The static nature of these early approaches made it difficult to incorporate new findings or adapt to the dynamic landscape of genomic research Tanisawa et al. (2020). While foundational, these methods struggled with large scale data and the discovery of novel patterns, prompting the need for more flexible approaches.

The introduction of machine learning marked a pivotal shift, enabling the analysis of large genomic datasets to uncover previously undetectable patterns Wang et al. (2024). Machine learning models, including supervised and unsupervised techniques, facilitated the automatic learning of relationships between genetic markers and athletic traits Munoz-Macho et al. (2024). For instance, clustering algorithms and support vector machines were used to classify athletes based on genetic profiles, while regression models predicted performance outcomes. Despite these advancements, machine learning required extensive feature engineering and domain expertise, and often fell short in capturing complex, hierarchical relationships within the data Yadav et al. (2025). This highlighted the need for more sophisticated methods capable of handling the intricate nature of genomic data.

The advent of deep learning and pre trained models has transformed genomic analysis in sports education, offering solutions to previous challenges Spanakis et al. (2025). Deep learning models, such as convolutional neural networks (CNNs) and recurrent neural networks (RNNs), excel in capturing complex patterns and hierarchical relationships within large scale genomic datasets. These models automatically learn features from raw data, reducing the need for extensive feature engineering Spanakis et al. (2024). Pre trained models, including transformer based architectures, facilitate knowledge transfer from large datasets to specific tasks related to athletic performance. Despite their success, deep learning models demand substantial computational resources and large labeled datasets, which can be difficult to obtain in sports genomics. Their black box nature can hinder interpretability, posing challenges in translating findings into actionable insights for sports education.

Based on the aforementioned limitations of existing methods, we propose a novel approach that leverages the strengths of deep learning while addressing its challenges to provide actionable genomic insights into athletic performance. Our method incorporates a hybrid framework that combines the interpretability of symbolic AI with the scalability and adaptability of deep learning. By integrating domain knowledge into the training process, our approach enhances the interpretability of deep learning models, enabling researchers and practitioners to better understand the genetic factors influencing athletic traits. Furthermore, our method employs advanced techniques for data augmentation and transfer learning to address the challenges of limited labeled data, ensuring robust performance across diverse genomic datasets. This hybrid approach not only bridges the gap between traditional and modern methodologies but also provides a comprehensive framework for advancing genomic research in sports education.

We summarize our contributions as follows:

- We propose a hybrid framework that integrates domain knowledge with deep learning to enhance interpretability and scalability in genomic analysis for athletic performance.
- Our method demonstrates high efficiency and generalizability, making it suitable for diverse genomic datasets and applicable across various sports education scenarios.
- Experimental results show that our approach outperforms existing methods in identifying genetic markers associated with athletic traits, providing actionable insights for personalized training and performance optimization.

## 2 Related work

### 2.1 Deep learning in genomic analysis

Deep learning has emerged as a transformative tool in genomic analysis, offering unprecedented capabilities in processing and interpreting complex biological data. Genomic data, characterized by its high dimensionality and intricate patterns, poses significant challenges for traditional computational methods Bottura and Correia (2025). Deep learning models, particularly convolutional neural networks (CNNs), recurrent neural networks (RNNs), and transformer based architectures, have demonstrated remarkable success in addressing these challenges. These models excel in identifying patterns, relationships, and features within genomic sequences, enabling researchers to uncover insights that were previously inaccessible. One of the primary applications of deep learning in genomic analysis is the prediction of gene expression levels and regulatory elements Yağcı (2022). By training on large scale genomic datasets, deep learning models can identify promoter regions, enhancers, and other regulatory sequences with high accuracy. This capability is crucial for understanding the genetic basis of traits, including those related to athletic performance. Specific genetic variants associated with muscle strength, endurance, and recovery can be identified through deep learning based genomic analysis, providing valuable insights into the genetic predispositions of athletes. Another significant area of application is the identification of single nucleotide polymorphisms (SNPs) and their associations with phenotypic traits. Deep learning models can analyze vast genomic datasets to pinpoint SNPs that correlate with athletic performance metrics, such as VO2 max, muscle fiber composition, and injury susceptibility Bulgay et al. (2024). These insights can inform personalized training regimens and injury prevention strategies, enhancing athletic performance and reducing the risk of overtraining. Furthermore, deep learning has been instrumental in advancing the field of epigenomics, which explores the influence of epigenetic modifications on gene expression. By integrating genomic and epigenomic data, deep learning models can uncover the complex interplay between genetic and environmental factors in shaping athletic performance. This holistic approach provides a more comprehensive understanding of the biological mechanisms underlying athletic traits, paving the way for targeted interventions and optimized training programs Carneiro et al. (2018).

### 2.2 Athletic performance prediction models

The application of deep learning in predicting athletic performance has gained significant traction in recent years. These models leverage diverse datasets, including genomic, physiological, and biomechanical data, to predict an athlete’s potential and optimize their training strategies. By integrating genomic insights with other performance related variables, deep learning models can provide a more nuanced understanding of the factors influencing athletic success Bakr et al. (2025). One of the key advantages of deep learning based performance prediction models is their ability to handle complex, non linear relationships between variables. Traditional statistical methods often struggle to capture these relationships, particularly when dealing with high dimensional data. Deep learning models, on the other hand, can learn intricate patterns and interactions within the data, enabling more accurate predictions of athletic performance outcomes Shen and Jing (2025). By analyzing genomic data alongside training load, nutrition, and recovery metrics, deep learning models can predict an athlete’s response to specific training interventions, helping coaches and sports scientists tailor their approaches to individual needs. Another important application of these models is talent identification and scouting. By analyzing genomic and performance data from a large pool of athletes, deep learning models can identify individuals with exceptional potential for success in specific sports. This approach not only enhances the efficiency of talent identification processes but also ensures that athletes are matched with sports that align with their genetic predispositions and physical attributes Yuan et al. (2025). Genomic markers associated with fast twitch muscle fibers may indicate a predisposition for sprinting or power based sports, while markers linked to slow twitch fibers may suggest suitability for endurance events. Injury prediction and prevention is another critical area where deep learning based performance models have shown promise. By analyzing genomic data in conjunction with biomechanical and physiological variables, these models can identify athletes at higher risk of specific injuries, such as ligament tears or stress fractures. This information can inform targeted interventions, such as strength training programs or biomechanical adjustments, to mitigate injury risks and enhance long term performance Saxena (2022).

Recent studies in sports genetics have further highlighted the importance of analyzing athletic performance within well characterized athlete populations and with explicit consideration of competitive level. Genome wide association evidence from high performance athlete cohorts has shown that genetic variants may be associated not only with performance related traits but also with injury related outcomes Ebert et al. (2023). In parallel, longitudinal studies in sport specific cohorts have demonstrated that polymorphisms such as ACE and ACTN3 can be meaningfully linked to endurance related capacity when the participants belong to a clearly defined athletic discipline Mägi et al. (2016). These findings suggest that the interpretation of genetic effects may depend strongly on sport discipline, competitive background, and athlete level, including whether participants compete at national or international standards. Compared with such athlete centered genetic association studies, the present work addresses a different problem setting, namely, cohort based prediction of general athletic capability from genotype marker representations and standardized physical measurements. Therefore, the proposed framework is intended to complement the existing sports genetics literature through population level predictive modeling, rather than replace sport specific or elite level genetic association studies.

### 2.3 Personalized training and nutrition

Personalized training and nutrition strategies have become a focal point in sports science, driven by the recognition that individual differences in genetics, physiology, and metabolism significantly influence athletic performance. Deep learning has played a pivotal role in advancing this field by enabling the integration and analysis of diverse datasets to develop tailored interventions for athletes. Genomic data provides a wealth of information about an individual’s genetic predispositions, including their response to training stimuli, recovery capacity, and nutritional requirements Zhang et al. (2025). Deep learning models can analyze this data to identify genetic variants associated with key performance traits, such as aerobic capacity, muscle strength, and injury susceptibility. By combining these insights with data on an athlete’s training history, physiological metrics, and dietary habits, deep learning models can generate personalized training and nutrition plans that optimize performance and minimize the risk of overtraining or injury Wang and Zhang (2024). One of the most significant applications of personalized training is the development of adaptive training programs. Deep learning models can continuously analyze an athlete’s performance data, adjusting training intensity, volume, and recovery periods in real time to ensure optimal progress. This dynamic approach accounts for individual variability in training responses, enabling athletes to achieve their performance goals more efficiently. Athletes with genetic markers indicating a slower recovery rate may benefit from longer rest periods between high intensity sessions, while those with markers for enhanced endurance capacity may thrive on higher training volumes Li and Li (2022). In the realm of nutrition, deep learning models can provide personalized dietary recommendations based on an athlete’s genetic profile, metabolic rate, and performance goals Görmez et al. (2025). Genetic variants associated with carbohydrate metabolism can inform the optimal macronutrient composition of an athlete’s diet, while markers linked to vitamin and mineral absorption can guide supplementation strategies. These personalized recommendations not only enhance performance but also support health and wellbeing, ensuring that athletes can sustain their training and competition schedules over the long term Le Goallec et al. (2023). Moreover, deep learning has facilitated the integration of wearable technology data into personalized training and nutrition strategies. By analyzing real time data from devices such as heart rate monitors, GPS trackers, and sleep trackers, deep learning models can provide actionable insights into an athlete’s physiological state and recovery status. This information can be used to fine tune training and nutrition plans, ensuring that athletes are consistently performing at their best while minimizing the risk of burnout or injury Görmez et al. (2025).

Although prior studies have demonstrated the effectiveness of deep learning and machine learning techniques in genomic analysis and athletic performance prediction, several limitations remain. Many existing genomic prediction models primarily rely on conventional regression or shallow machine learning algorithms. While these approaches can capture simple linear or low order nonlinear relationships, they often struggle to model the high dimensional and hierarchical interactions among genetic markers. Genomic features such as SNPs exhibit complex linkage disequilibrium structures and epistatic interactions, which are difficult to fully represent using traditional feature engineering or tree based ensemble methods. As a result, predictive performance may be limited, particularly when modeling subtle genotype phenotype relationships. Although deep learning models have improved representation learning capability, many existing studies treat genomic features as generic tabular inputs without explicitly incorporating biological structure or domain constraints Wu et al. (2021). The absence of biologically informed regularization may lead to overfitting or spurious correlations, especially when the number of genomic features greatly exceeds the number of samples. Without structural guidance, learned latent representations may lack interpretability and biological plausibility. Uncertainty estimation is often overlooked in athletic performance prediction models. Most approaches focus solely on point predictions without quantifying predictive confidence Alharbi and Rashid (2022). However, genomic data inherently contain measurement noise, population heterogeneity, and incomplete phenotype information. Ignoring uncertainty may reduce model reliability and limit practical applicability in sports education contexts, where decision making requires not only accuracy but also confidence awareness. Existing methods typically optimize predictive accuracy alone, without explicitly enforcing consistency between genomic representations and domain knowledge. In the absence of constraint aware learning mechanisms, models may produce predictions that are statistically accurate yet biologically implausible Nwabuwe (2025). This gap highlights the need for approaches that integrate representation learning with domain specific constraints. These limitations motivate the development of a structured framework that combines manifold informed representation learning, constraint based optimization, and uncertainty aware prediction. By embedding genomic features into biologically consistent latent spaces and explicitly modeling predictive variance, the proposed method aims to enhance both predictive accuracy and interpretability while maintaining robustness across diverse cohort datasets.

## 3 Methods

### 3.1 Overview

The proposed methodology employs advanced deep learning techniques to extract genomic insights pertinent to athletic performance within the realm of sports education. This section delineates the framework, which is meticulously crafted to tackle the complexities inherent in modeling genomic data and its correlation with athletic capabilities. The methodology is systematically divided into three principal components: *Preliminaries* Section 3.2, *Genomic Athletic Predictor* Section 3.3, and *Constrained Optimization Refinement and Uncertainty Aware Prediction* Section 3.4. Each component is integral to the framework, ensuring a methodical approach to the problem at hand.

In *Preliminaries* Section 3.2, the mathematical underpinnings are established, and the problem of predicting athletic performance from genomic data is formalized. This involves defining the genomic feature space, athletic performance metrics, and elucidating the interrelationships among these variables. The section introduces the necessary notations and constructs, providing the foundation for the subsequent components of the methodology. By symbolically representing the problem, clarity and precision are achieved, facilitating the development of a robust predictive framework.

The *Genomic Athletic Predictor* Section 3.3 serves as the cornerstone of the proposed methodology. This innovative model integrates three specialized modules: the *Manifold Informed Constraint Encoder*, the *Agent Driven Genomic Planner*, and the *Uncertainty Guided Athletic Forecaster*. Each module is meticulously designed to address specific challenges associated with genomic data analysis and athletic performance prediction. The *Manifold Informed Constraint Encoder* is tasked with embedding genomic data into a meaningful latent space while maintaining structural constraints. The *Agent Driven Genomic Planner* introduces a dynamic planning mechanism to optimize the representation of genomic features in relation to athletic performance. The *Uncertainty Guided Athletic Forecaster* incorporates uncertainty quantification to enhance prediction reliability. Collectively, these modules form a cohesive model that captures the intricate relationships between genomic data and athletic outcomes.

The final component, *Constrained Optimization Refinement and Uncertainty Aware Prediction* Section 3.4, articulates the strategic approach employed to refine the predictive capabilities of the *Genomic Athletic Predictor*. This section delves into the optimization techniques used to enforce constraints derived from domain knowledge, ensuring the model adheres to biologically plausible relationships. It underscores the significance of uncertainty aware prediction in addressing the inherent variability and noise present in genomic data. By integrating uncertainty into the prediction process, the methodology aims to provide actionable insights with a heightened degree of confidence, which is crucial for applications in sports education.

### 3.2 Preliminaries

The task of deriving genomic insights into athletic performance within sports education is approached by formalizing the problem and establishing a mathematical framework. The aim is to predict athletic performance metrics based on genomic data, incorporating constraints informed by manifold structures and addressing prediction uncertainty. This subsection introduces the notation, problem formulation, and foundational concepts that underpin the proposed methodology.

Let $G=\left\{g_{1},g_{2},\ldots ,g_{n}\right\}$ denote the genomic feature space, where $g_{i}$ represents the i -th genomic feature extracted from an athlete. These features are derived from genomic sequencing and preprocessing pipelines, capturing genetic markers associated with athletic performance. The athletic performance metrics are denoted as $A=\left\{a_{1},a_{2},\ldots ,a_{m}\right\}$ , where $a_{j}$ represents the j -th performance metric, such as endurance, strength, or speed.

The relationship between genomic features G and athletic performance metrics A is modeled as a mapping function f:G→A , parameterized by a deep learning model. The objective is to learn f such that the predicted performance metrics $\hat{A}=f\left(G\right)$ closely approximate the true metrics A , while adhering to domain specific constraints and accounting for uncertainty.

To incorporate manifold informed constraints, it is assumed that the genomic feature space G lies on a lower dimensional manifold $M\subset R^{n}$ . This assumption is motivated by the observation that genomic data often exhibit intrinsic structures due to biological processes. Let Φ:G→M be a mapping that projects genomic features onto the manifold M . The manifold structure is characterized by a constraint function C:M→R , which encodes domain specific relationships and ensures that the projected features Φ(G) satisfy these constraints. Mathematically, this can be expressed as Equation 1:

$$C\left(\Phi \left(G\right)\right)=0,$$ (1)

where C is designed to enforce biologically plausible relationships among genomic features.

Uncertainty in predictions is modeled using a probabilistic framework. Let $U=\left\{u_{1},u_{2},\ldots ,u_{m}\right\}$ represent the uncertainty associated with each predicted performance metric ${\hat{a}}_{j}$ . The uncertainty aware prediction model aims to estimate both the mean ${\hat{a}}_{j}$ and the uncertainty $u_{j}$ for each metric. This is achieved by defining the predictive distribution $p\left(a_{j}|G\right)$ , which captures the likelihood of observing $a_{j}$ given the genomic features G . The predictive distribution is parameterized as Equation 2:

$$p\left(a_{j}|G\right)=N\left({\hat{a}}_{j},u_{j}^{2}\right),$$ (2)

where N denotes a Gaussian distribution with mean ${\hat{a}}_{j}$ and variance $u_{j}^{2}$ .

The optimization problem is formulated as a constrained learning task. Let $L\left(\hat{A},A\right)$ denote the loss function that quantifies the discrepancy between predicted and true performance metrics. The learning objective is to minimize L while satisfying the manifold informed constraints and accounting for uncertainty. This can be expressed as Equation 3:

$$\underset{f}{\min}L\left(\hat{A},A\right)\;\text{subject to}\;C\left(\Phi \left(G\right)\right)=0\;\text{and}\;p\left(a_{j}|G\right)=N\left({\hat{a}}_{j},u_{j}^{2}\right).$$ (3)

To facilitate optimization, a regularization term R(Φ(G)) is introduced to penalize deviations from the manifold structure. The regularized loss function is given by Equation 4:

$$L_{\text{reg}}=L\left(\hat{A},A\right)+\lambda R\left(\Phi \left(G\right)\right),$$ (4)

where λ is a hyperparameter controlling the trade off between prediction accuracy and adherence to the manifold constraints.

The problem is formalized as learning a mapping f that predicts athletic performance metrics A from genomic features G , while satisfying manifold informed constraints and modeling uncertainty. The subsequent sections will detail the proposed Genomic Athletic Predictor model and the constrained optimization refinement strategy that address these challenges.

### 3.3 Genomic athletic predictor

**FIGURE 1:**
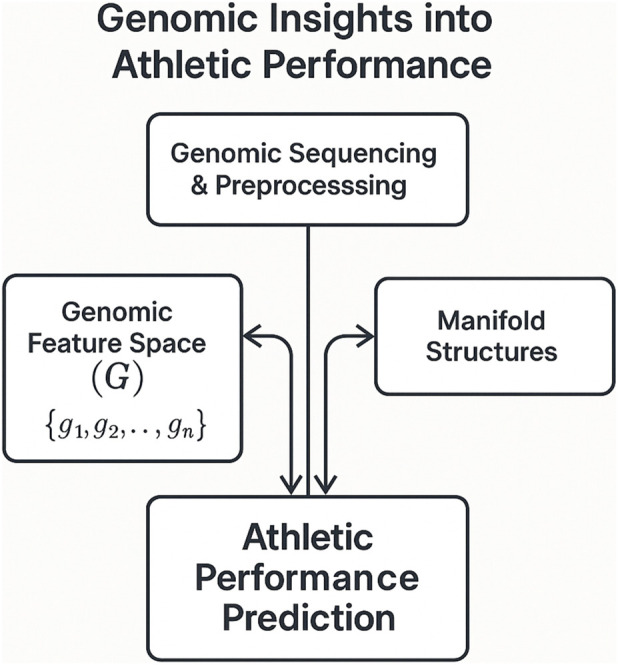
This figure illustrates key aspects of the methodology described in the subsection.

The Genomic Athletic Predictor is a sophisticated model designed to harness deep learning techniques for extracting genomic insights that contribute to understanding athletic performance in sports education Figure 1. This model integrates three pivotal modules: the Manifold Informed Constraint Encoder, the Agent Driven Genomic Planner, and the Uncertainty Guided Athletic Forecaster. Each module is meticulously crafted to address specific challenges in genomic data analysis and athletic performance prediction, ensuring a comprehensive and robust framework.

#### 3.3.1 Manifold informed constraint encoder

**FIGURE 2:**
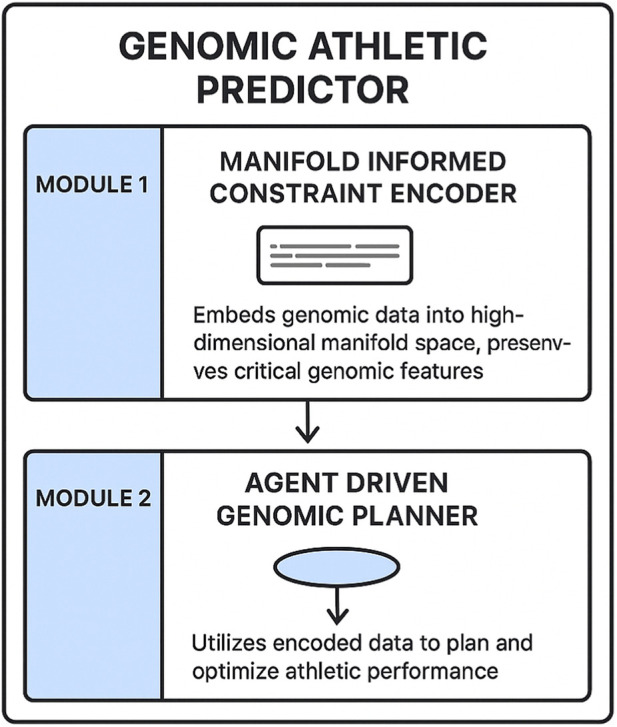
The Genomic Athletic Predictor comprises two key modules: the Manifold Informed Constraint Encoder and the Agent Driven Genomic Planner. The Manifold Informed Constraint Encoder embeds genomic data into a high dimensional manifold space, ensuring the preservation of critical genomic features. Subsequently, the Agent Driven Genomic Planner utilizes the encoded data to plan and optimize athletic performance, translating genomic insights into actionable strategies.

The Genomic Athletic Predictor begins with the Manifold Informed Constraint Encoder, which is responsible for embedding genomic data into a high dimensional manifold space while preserving critical structural and biological constraints Figure 2. Let $X\in R^{n\times d}$ represent the genomic feature matrix, where n denotes the number of samples and d the dimensionality of the genomic features. The encoder maps X into a latent space $Z\in R^{n\times k}$ , where k is the reduced dimensionality, using a transformation function $f_{\text{enc}}:R^{d}\to R^{k}$ . This transformation is defined as Equation 5:

$$Z=f_{\text{enc}}\left(X;\Theta_{\text{enc}}\right)$$ (5)

where $\Theta_{\text{enc}}$ represents the learnable parameters of the encoder. To ensure the manifold constraints are respected, a penalty term $L_{\text{manifold}}$ is introduced, which enforces the preservation of genomic relationships (Equation 6):

$$L_{\text{manifold}}=\underset{i,j}{\sum }\|Z_{i}-Z_{j}{\|}^{2}\cdot C_{ij}$$ (6)

Here, $C_{ij}$ encodes the biological similarity between samples i and j , derived from domain specific genomic metrics. This module ensures that the embedded genomic data maintains its inherent biological structure, which is crucial for accurate downstream analysis.

#### 3.3.2 Agent driven genomic planner

The second module, the Agent Driven Genomic Planner, operates on the latent representation Z to identify actionable genomic patterns that influence athletic performance. This module employs a planning function $f_{\text{plan}}:R^{k}\to R^{m}$ , where m represents the number of actionable insights. The planner generates a genomic action matrix $A\in R^{n\times m}$ (Equation 7):

$$A=f_{\text{plan}}\left(Z;\Theta_{\text{plan}}\right)$$ (7)

The planning process is guided by a reward function R(A) , which quantifies the effectiveness of the genomic actions in enhancing athletic performance (Equation 8):

$$R\left(A\right)=\overset{n}{\underset{i=1}{\sum }}\phi \left(A_{i},y_{i}\right)$$ (8)

where ϕ is a domain specific reward function and $y_{i}$ represents the observed athletic performance metrics for sample i . This module is crucial for translating genomic data into actionable insights that can directly impact athletic performance, providing a bridge between genomic information and practical applications in sports education.

#### 3.3.3 Uncertainty guided athletic forecaster

The Uncertainty Guided Athletic Forecaster predicts athletic performance while accounting for uncertainties inherent in genomic data. This module employs a probabilistic forecasting function $f_{\text{forecast}}:R^{m}\to R^{p}$ , where p denotes the dimensionality of the performance metrics. The forecaster outputs a prediction matrix $P\in R^{n\times p}$ (Equation 9):

$$P=f_{\text{forecast}}\left(A;\Theta_{\text{forecast}}\right)$$ (9)

To model uncertainty, the forecaster incorporates a variance term $\sigma^{2}$ for each prediction (Equation 10):

$$P_{i}=\mu_{i}+\sigma_{i}^{2}$$ (10)

where $\mu_{i}$ is the mean prediction and $\sigma_{i}^{2}$ represents the uncertainty. The uncertainty is minimized using a loss function $L_{\text{uncertainty}}$ (Equation 11):

$$L_{\text{uncertainty}}=\overset{n}{\underset{i=1}{\sum }}\sigma_{i}^{2}$$ (11)

This module is essential for providing reliable predictions of athletic performance, taking into account the inherent variability and uncertainty in genomic data, thus enhancing the robustness and accuracy of the model’s predictions.

The Genomic Athletic Predictor is trained end to end by optimizing a composite loss function (Equation 12):

$$L_{\text{total}}=L_{\text{manifold}}+L_{\text{reward}}+L_{\text{uncertainty}}$$ (12)

where $L_{\text{reward}}$ quantifies the alignment between genomic actions and observed performance metrics. The model’s architecture ensures that genomic insights are effectively utilized to predict athletic performance while addressing the challenges of high dimensionality, biological constraints, and uncertainty. The integration of these modules provides a novel framework for genomic analysis in sports education, achieving a balance between biological fidelity and predictive accuracy, paving the way for actionable insights into athletic performance.

### 3.4 Uncertainty aware prediction

**FIGURE 3:**
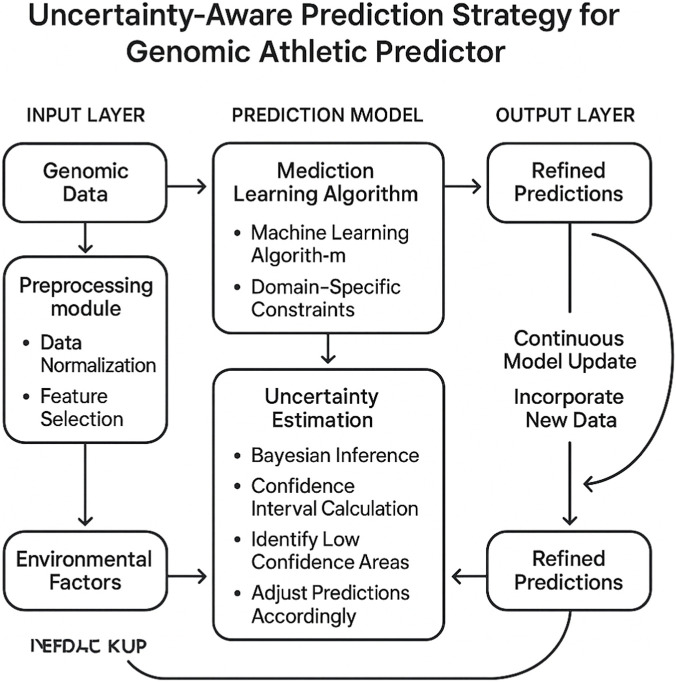
The diagram illustrates the uncertainty aware prediction strategy for the Genomic Athletic Predictor. It highlights the integration of genomic data and environmental factors through a preprocessing module, followed by a prediction model that incorporates a mediation learning algorithm and domain specific constraints. Uncertainty estimation is achieved via Bayesian inference and confidence interval calculations, enabling the identification of low confidence areas and adjustment of predictions. The strategy supports continuous model updates by incorporating new data to refine predictions.

In this subsection, we elaborate on the proposed uncertainty aware prediction strategy, which is designed to enhance the robustness and interpretability of the Genomic Athletic Predictor Figure 3. This strategy leverages domain specific constraints and uncertainty quantification to refine predictions, ensuring that the model not only achieves high accuracy but also provides actionable insights into the genomic factors influencing athletic performance. By integrating uncertainty estimation into the prediction pipeline, the model is capable of identifying areas of low confidence, which is critical for applications in sports education where decisions often have significant implications.

#### 3.4.1 Uncertainty quantification and propagation

The core idea of the uncertainty aware prediction strategy is to model and propagate uncertainty throughout the prediction process. Let $X\in R^{n\times d}$ represent the genomic feature matrix, where n is the number of samples and d is the dimensionality of the genomic features. The target variable, athletic performance, is denoted as $y\in R^{n}$ . The Genomic Athletic Predictor aims to learn a mapping function $f:R^{d}\to R$ such that $\hat{y}=f\left(X\right)$ minimizes the prediction error. However, instead of directly optimizing for $\hat{y}$ , we introduce an uncertainty term $\sigma^{2}\in R^{n}$ to capture the model’s confidence in its predictions.

The predictive distribution is modeled as Equation 13:

$$p\left(y|X\right)=N\left(\hat{y},\text{diag}\left(\sigma^{2}\right)\right),$$ (13)

where N denotes a Gaussian distribution with mean $\hat{y}$ and variance $\sigma^{2}$ . The variance $\sigma^{2}$ is learned alongside the predictive mean $\hat{y}$ , allowing the model to quantify uncertainty in its predictions.

To incorporate uncertainty into the optimization process, we define a loss function that combines the prediction error and the uncertainty penalty (Equation 14):

$$L=\frac{1}{n}\overset{n}{\underset{i=1}{\sum }}\left(\frac{{\left(y_{i}-{\hat{y}}_{i}\right)}^{2}}{2\sigma_{i}^{2}}+\frac{1}{2}\log\sigma_{i}^{2}\right),$$ (14)

where $y_{i}$ and ${\hat{y}}_{i}$ are the true and predicted values for the i -th sample, respectively, and $\sigma_{i}^{2}$ is the predicted variance for the i -th sample. The first term penalizes large prediction errors, while the second term encourages the model to avoid overestimating uncertainty.

#### 3.4.2 Domain specific constraints integration

To further refine the predictions, we incorporate domain specific constraints derived from the manifold informed constraint encoder. Let $C\left(X,\hat{y}\right)$ represent a set of constraints that encode prior knowledge about the relationships between genomic features and athletic performance. These constraints are enforced through a penalty term (Equation 15):

$$L_{\text{constraint}}=\lambda \cdot C\left(X,\hat{y}\right),$$ (15)

where λ is a hyperparameter that controls the strength of the constraint penalty. The total loss function becomes Equation 16:

$$L_{\text{total}}=L+L_{\text{constraint}}.$$ (16)

#### 3.4.3 Bayesian framework for robustness

The uncertainty aware prediction strategy also incorporates a Bayesian framework to further enhance robustness. By treating the model parameters θ as random variables, we define a posterior distribution p(θ|X,y) that captures the uncertainty in the parameter estimates. Using Bayes’ theorem, the posterior is given by Equation 17:

$$p\left(\theta |X,y\right)\propto p\left(y|X,\theta \right)p\left(\theta \right),$$ (17)

where p(θ) is the prior distribution over the parameters. The predictive distribution is then obtained by marginalizing over the posterior (Equation 18):

$$p\left(y^{*}|X^{*},X,y\right)=\int p\left(y^{*}|X^{*},\theta \right)p\left(\theta |X,y\right)d\theta ,$$ (18)

where $X^{*}$ and $y^{*}$ denote the test inputs and outputs, respectively. In practice, this integral is approximated using techniques such as Monte Carlo sampling or variational inference.

To ensure that the uncertainty estimates are reliable, we employ a calibration procedure. Let $\hat{F}\left(y\right)$ denote the empirical cumulative distribution function (CDF) of the predicted values, and F(y) the true CDF. The calibration error is defined as Equation 19:

$$\text{Calibration Error}=\int_{-\infty }^{\infty }|\hat{F}\left(y\right)-F\left(y\right)|dy.$$ (19)

Minimizing this error ensures that the predicted uncertainties align with the true uncertainties, thereby improving the interpretability of the model.

The uncertainty aware prediction strategy is integrated with the agent driven genomic planner to provide actionable insights. By analyzing the uncertainty estimates, the planner can identify genomic features that contribute most to the uncertainty, guiding future data collection and model refinement efforts. This iterative process ensures that the Genomic Athletic Predictor remains adaptive and continues to improve as more data becomes available.

### 3.5 Advantages and innovations of the proposed framework

The proposed framework is developed to systematically address the key limitations observed in existing genomic based athletic performance prediction methods. Rather than relying solely on conventional regression or purely data driven deep learning architectures, the framework integrates structured representation learning, domain informed constraints, and uncertainty aware modeling within a unified optimization paradigm. To effectively model the high dimensionality and complex genotype phenotype interactions inherent in genomic data, the framework introduces a Manifold Informed Constraint Encoder that embeds genomic features into a structured latent manifold space. This approach captures intrinsic correlations among genetic markers while reducing redundant interactions and noise. By preserving biologically meaningful relationships during representation learning, the model enhances generalization capability and mitigates the overfitting risks commonly observed in high dimensional genomic prediction tasks. In contrast to prior approaches that directly map genomic vectors to performance scores, the framework incorporates an Agent Driven Genomic Planner that introduces an intermediate structured representation between latent genomic features and athletic outcomes. This hierarchical modeling mechanism strengthens the semantic linkage between genotype patterns and predicted performance components, improving interpretability and enabling a clearer understanding of how genomic information contributes to athletic capability. The integration of an Uncertainty Guided Athletic Forecaster enables the model to move beyond deterministic point estimation. By jointly predicting performance values and associated variances within a probabilistic framework, the method explicitly quantifies predictive confidence. This uncertainty aware design enhances robustness when handling noisy genomic measurements, heterogeneous cohort distributions, and incomplete phenotype information, thereby supporting more reliable decision making in sports education contexts. The framework also incorporates constrained optimization refinement to ensure that learned representations remain consistent with biologically plausible relationships. By embedding domain specific constraints directly into the training objective, the model reduces the likelihood of spurious correlations and improves biological fidelity. This integration of constraint based regularization differentiates the proposed approach from purely data driven models that lack structural guidance. Through the combined effects of structured manifold embedding, hierarchical genotype phenotype modeling, uncertainty aware prediction, and biologically informed constraint enforcement, the proposed framework achieves improved predictive accuracy, enhanced robustness, and greater interpretability compared to existing methods. These advantages position the approach as a comprehensive and reliable solution for genomic driven athletic performance prediction in sports education.

## 4 Experiments

### 4.1 Task definition

The task studied in this work is individual athletic potential prediction under a supervised learning setting. The input variable X consists of two components: student genomic feature data and historical athletic performance records. Genomic features are represented as high dimensional genotype vectors describing inherited biological characteristics. Historical athletic performance records include measured indicators of physical capability such as speed, endurance, and strength obtained from standardized physical tests. These heterogeneous features are processed through the proposed framework to learn the mapping from genomic and historical physiological attributes to future athletic capability. The output variable Y is a continuous athletic potential score that quantitatively represents the predicted athletic performance capacity of each individual. The task is formulated as a regression problem where the model estimates a scalar potential score for each student based on the joint representation of genetic information and prior athletic indicators.

The supervision signal is derived from empirical athletic performance measurements collected within large scale cohort studies. The ground truth labels correspond to standardized physical performance indicators recorded through controlled athletic testing protocols in the cohort datasets. These measurements include quantitative assessments of physical capability such as running speed, endurance capacity, and muscular strength, which are systematically collected as part of the cohort data acquisition process. The athletic potential score used as the training target is constructed from these recorded performance statistics through standardized normalization procedures to ensure comparability across individuals. Therefore, the supervision signal originates from systematically collected cohort statistics rather than manual annotation, providing reliable and objective labels for supervised regression learning.

It should be emphasized that the prediction task studied in this work is not an elite athlete ranking task and is not designed to identify national team or international level competitors. Instead, the task is formulated on population based cohort participants for whom paired genotype features and standardized physical capability measurements are available. In the publicly accessible datasets used in this study, the available non genomic variables are limited to structured physical performance records and aligned cohort phenotypes used to construct the athletic potential score. Detailed clinical diagnosis variables, sport specific participation labels, competition level annotations, and systematic injury history records are not consistently available in the processed data used for model development. Therefore, the present framework should be interpreted as a population level athletic capability prediction model based on genomic and standardized physical assessment data, rather than a direct predictor of elite competitive achievement. In the present study, the genetic input is restricted to genotype level inherited marker information represented as high dimensional numerical genotype vectors. The model does not use other molecular layers such as transcriptomic profiles, epigenetic measurements, copy number variation data, proteomic assays, or polygenic risk scores. Therefore, the genomic component of the input should be interpreted as a structured genotype marker representation derived from cohort genotyping resources. The output of the present framework should be interpreted as a continuous athletic potential score derived from standardized physical capability measurements rather than as a direct prediction of competitive results, injury occurrence, or career duration. Accordingly, the practical role of the model in its current form is to support data driven assessment of general athletic capability and training related physical potential within cohort based or sports education settings. It is not designed to forecast match outcomes, medal rankings, injury timelines, or the length of an athlete’s professional career.

### 4.2 Dataset and data preprocessing

#### 4.2.1 Datasets

This study evaluates the proposed framework using two publicly accessible cohort datasets that contain paired genomic and physical performance measurements. The first dataset is the Avon Longitudinal Study of Parents and Children (ALSPAC) cohort, a population study that records genomic and physiological development data. After applying the filtering rules described below, the dataset contains 7,412 individuals with complete genotype features and standardized athletic performance measurements. Each sample includes a high dimensional genomic feature vector derived from genotyping arrays and three standardized physical capability indicators consisting of running speed, endurance capacity, and muscular strength. The supervision labels are obtained from the standardized physical fitness testing protocol used in the cohort study, where trained research staff collect measurements following unified testing procedures. Records that contain incomplete genotype information or missing athletic performance measurements are removed to ensure data consistency. The dataset is randomly divided into training, validation, and test sets with a ratio of 8:1:1, and the split is performed at the individual level to ensure that each participant appears in only one partition. The second dataset is the TwinsUK cohort dataset, a longitudinal adult twin registry containing genetic information and physical capability measurements. After data cleaning and filtering, the dataset includes 5,036 individuals with paired genotype features and athletic test results. Each record contains genomic markers extracted from the genotyping platform together with standardized measurements of speed, endurance, and strength collected during controlled physical examinations. The supervision signal is derived directly from these recorded athletic performance statistics. Samples with missing genotype markers, incomplete athletic test records, or inconsistent measurement entries are removed during preprocessing. The processed dataset is divided using the same 8:1:1 random split strategy for training, validation, and testing. This consistent splitting protocol ensures reliable model evaluation and prevents information leakage between experimental partitions.

To clarify the scope of the datasets, the information used in this study can be grouped into two categories: genomic data and standardized physical capability data. The genomic component consists of genotype derived numerical feature vectors obtained from the cohort genotyping platforms after preprocessing and quality filtering. The athletic component consists of standardized measurements of running speed, endurance capacity, and muscular strength collected under cohort testing protocols. These variables are used to construct the continuous athletic potential score for supervised regression. The public cohort resources employed in this study do not provide a unified annotation of sport discipline, training specialization, or competition level for all retained individuals. Consequently, the participants analyzed in this work should not be interpreted as exclusively elite athletes, national team athletes, or international level competitors. No systematic injury status variable or injury history annotation was included in the final modeling pipeline. For this reason, the present experiments evaluate genomic associations with general physical performance capacity rather than sport specific expertise, elite competitive status, or injury related outcomes. The genetic data used in this work consist of genotype derived marker features obtained from cohort genotyping platforms. For each retained individual, the available inherited genetic information is represented as a high dimensional marker vector derived from array based or platform based genotyping records after quality filtering. These markers are used as the genomic input to the prediction model in numerical vector form. No additional omics layers, such as whole transcriptome expression, DNA methylation profiles, copy number variation measurements, proteomic data, or externally constructed polygenic scores, were included in the present modeling pipeline. Accordingly, the proposed framework operates on genotype marker representations rather than multimodal molecular profiles.

#### 4.2.2 Data preprocessing

The raw cohort data obtained from the ALSPAC dataset Jamieson et al. (2025) and the TwinsUK dataset Watanabe et al. (2023) are processed through a standardized preprocessing pipeline to ensure data quality and consistency. Data cleaning first removes duplicate records according to the unique participant identifier provided by each cohort. Samples containing missing genotype markers or incomplete athletic performance measurements are removed to guarantee that each record contains both genomic features and physical performance indicators. Outlier detection is conducted on athletic measurements using a three standard deviation rule. Records with any performance value exceeding three standard deviations from the cohort mean are treated as abnormal measurements and are removed from the dataset. Genotype features are also filtered by removing markers with missing values to maintain consistent feature dimensionality across individuals. After filtering, the retained genotype markers are encoded into numerical genotype vectors that can be processed by the deep learning framework. These genotype derived marker vectors are then normalized to the interval [0,1] to provide a stable and consistent input representation across individuals and across the two cohort datasets. This preprocessing step ensures that the model operates on harmonized genotype level features rather than raw platform specific genotype records.

After data cleaning, feature standardization is applied to align heterogeneous attributes. Genomic features are transformed into numerical genotype vectors and normalized using min–max normalization to map each feature into the interval [0,1]. Athletic performance indicators including speed, endurance, and strength are standardized using z score normalization so that each variable has zero mean and unit variance across the training set. Identity alignment is performed using the unique participant identifier to guarantee that genomic data and athletic test records correspond to the same individual. The processed genomic vectors and standardized athletic indicators are concatenated to form the unified feature matrix X . This normalized representation provides stable numerical input for the manifold mapping module and ensures consistent training behavior across both cohort datasets.

### 4.3 Evaluation metrics and baseline

#### 4.3.1 Metrics definition

The performance of the proposed model is evaluated using four primary regression metrics and two efficiency metrics. The primary metrics measure the quality of athletic potential prediction. Pearson correlation coefficient evaluates the linear relationship between the predicted athletic potential score and the observed physical performance indicators. A higher correlation value indicates stronger consistency between predictions and empirical athletic measurements. Root Mean Square Error measures the average magnitude of prediction errors by computing the square root of the mean squared difference between predicted scores and ground truth performance statistics. The coefficient of determination *R* ^2^ quantifies the proportion of variance in the observed athletic performance that is explained by the predictive model. Mean Absolute Error measures the average absolute difference between predicted athletic potential scores and observed performance indicators, providing an interpretable measure of prediction deviation. To prediction accuracy, computational efficiency is evaluated through two resource oriented metrics. Model parameter count measures the total number of learnable parameters used by each model, reflecting storage and memory requirements. Floating Point Operations measures the computational complexity required for inference and indicates the computational cost of model deployment.

#### 4.3.2 Evaluation protocol

The evaluation protocol follows a standardized offline regression evaluation procedure using fixed dataset partitions. Each model is trained on the training split and optimized using the validation split for hyperparameter selection. The final evaluation is conducted on the held out test split to measure generalization performance. The prediction target is the athletic potential score constructed from standardized athletic performance indicators including speed, endurance, and strength. Model predictions are compared with the corresponding ground truth performance statistics in the test set. All evaluation metrics are computed over the entire test set to provide a global assessment of prediction quality. The protocol evaluates the mapping from genomic features and historical athletic performance records to future athletic capability. The same training, validation, and test partitions are used for all compared models to ensure fair comparison. The evaluation strictly measures performance under identical data preprocessing and feature representations, allowing a consistent comparison between traditional regression methods, machine learning models, and deep learning architectures.

#### 4.3.3 Statistical settings

To ensure reliable experimental conclusions, all models are trained and evaluated using repeated independent runs with different random initialization seeds. Each experiment is repeated ten times to reduce the effect of stochastic optimization and random data shuffling. For each evaluation metric, the mean value and standard deviation across the ten runs are reported to describe the central tendency and performance variability. This repeated evaluation protocol provides a stable estimation of model performance and ensures that reported results are not dependent on a single training instance. Statistical significance testing is conducted to compare the proposed method with baseline models. A two sided paired t-test is used to assess whether the observed performance differences between models are statistically significant. The test is performed on the metric values obtained from the repeated runs. A significance level of p < 0.05 is used to determine whether the improvement of the proposed method over the baseline models is statistically meaningful.

#### 4.3.4 Baseline

Six baseline models are selected to provide a comprehensive comparison across traditional regression methods, ensemble learning algorithms, neural network architectures, and lightweight models. Linear Regression is included as a classical statistical regression approach that models a linear relationship between genomic features and athletic performance indicators. Random Forest is used as a tree based ensemble learning method that captures nonlinear relationships through an aggregation of decision trees. Gradient Boosting Machine is included as a boosting based ensemble model that sequentially improves performance by reducing residual errors. Multilayer Perceptron represents a widely used deep neural network architecture for tabular regression tasks and provides a strong deep learning baseline. TabTransformer is selected as a state of the art deep learning model designed for tabular data representation learning using attention mechanisms. LightGBM is included as a lightweight gradient boosting framework that achieves efficient training and inference while maintaining strong performance on structured data.

### 4.4 Implementation details

All experiments are conducted on a workstation equipped with an Intel Xeon Silver 4314 CPU, an NVIDIA RTX 3090 GPU with 24 GB memory, and 128 GB RAM running Ubuntu 22.04 LTS. The implementation is developed using Python with PyTorch 2.1 as the primary deep learning framework. CUDA 12.1 and cuDNN 8.9 are used to accelerate GPU computation. Core scientific libraries include NumPy 1.26 for numerical operations, SciPy 1.11 for statistical computation, and scikit learn 1.3 for baseline implementations and preprocessing utilities. The preprocessing and augmentation of genomic data are performed using methods appropriate for structured one dimensional biological data. Genotype features are transformed into numerical vectors and normalized to the [0,1] interval using min–max normalization. Athletic performance indicators, including speed, endurance, and strength, are standardized using z score normalization across the training set. Outlier detection is applied to remove records exceeding three standard deviations from the mean for any performance metric. To evaluate robustness, small Gaussian noise is injected into genomic features, simulating measurement variability. These procedures ensure that all preprocessing and augmentation steps are compatible with structured genomic data and maintain biological consistency. Training follows a unified configuration across all experiments. Each model is trained for 120 epochs with a batch size of 64. The Adam optimizer is used with an initial learning rate of 0.001 and weight decay set to 0.0001 to stabilize parameter updates. A cosine annealing learning rate scheduler is applied to gradually decrease the learning rate during training. All experiments are initialized with a fixed random seed of 42 to ensure reproducibility across repeated runs. Model parameters are updated through backpropagation using mini batch gradient descent, and validation performance is monitored after each epoch to ensure stable convergence during training. The proposed model contains several structural hyperparameters that determine the architecture of the genomic prediction framework. The genomic feature encoder maps the input genotype vector into a latent manifold representation using a fully connected embedding layer with an embedding dimension of 128. The manifold informed constraint encoder consists of three stacked feedforward layers with hidden dimensions of 256, 128, and 64, each followed by layer normalization and ReLU activation to stabilize representation learning. The agent driven genomic planner transforms the latent representation into an action matrix using a two layer multilayer perceptron with hidden dimension 128 and output dimension corresponding to the predicted athletic capability components. The uncertainty guided athletic forecaster predicts the athletic potential score and associated variance using a dual head regression structure where the mean predictor and variance predictor share the same latent representation. The Bayesian inference component estimates predictive distributions using Monte Carlo sampling with 20 forward passes during inference. The constrained optimization module integrates manifold constraints through a regularization coefficient of 0.1 to maintain biological consistency during training. All baseline models are trained and evaluated under identical experimental conditions. Dataset partitions, preprocessing pipelines, and evaluation protocols are consistent across all methods. Hyperparameters of baseline models are tuned using the validation set while maintaining the same computational environment and training procedure to ensure fair and reproducible comparisons.

### 4.5 Results and discussion

#### 4.5.1 Comparative experiments

This section presents comparative experiments designed to evaluate the effectiveness of the proposed framework for athletic potential prediction. The evaluation is conducted on two cohort datasets containing paired genomic features and standardized athletic performance measurements. Six baseline methods are included for comparison: Linear Regression Homma et al. (2022), Random Forest Xu et al. (2025), Gradient Boosting Machine Chen et al. (2025), Multilayer Perceptron Thomas et al. (2025), TabTransformer Fan and Waldmann (2024), and LightGBM Mănescu and Mănescu (2025). These baselines cover classical statistical regression models, ensemble learning algorithms, neural network architectures, and lightweight gradient boosting approaches. Model performance is assessed using four primary regression metrics: Pearson correlation coefficient, Root Mean Square Error, coefficient of determination *R* ^2^, and Mean Absolute Error. These metrics evaluate prediction accuracy and the consistency between predicted athletic potential scores and observed physical performance measurements. Computational efficiency is analyzed through the number of learnable parameters and the number of floating point operations. All models are trained using identical data preprocessing procedures, dataset partitions, and evaluation protocols. Hyperparameters for baseline models are selected using the validation split, and all experiments are repeated multiple times with different random seeds to ensure statistical reliability and fair comparison.

Table 1 reports the performance of all compared methods on the ALSPAC cohort dataset. Classical statistical regression shows the weakest performance. Linear Regression achieves a Pearson correlation of 0.61 and an $R^{2}$ score of 0.37, indicating that simple linear relationships are insufficient for capturing the complex interactions between genomic features and athletic performance indicators. Ensemble learning models provide clear improvements. Random Forest increases the Pearson correlation to 0.69 and reduces RMSE from 0.347 to 0.311, while Gradient Boosting Machine further improves the correlation to 0.72 and reduces RMSE to 0.298. These improvements demonstrate that nonlinear feature interactions within genomic data are important for athletic potential prediction. Deep neural models show additional gains due to stronger representation learning capability. The Multilayer Perceptron achieves a Pearson correlation of 0.74 and an $R^{2}$ score of 0.52, outperforming traditional machine learning methods by learning higher level feature representations from high dimensional genomic inputs. TabTransformer further improves the correlation to 0.76 and reduces RMSE to 0.276, demonstrating the effectiveness of attention based feature interaction modeling for structured genomic data. LightGBM performs competitively with a Pearson score of 0.75 while maintaining relatively low error values. The proposed method achieves the best performance across all evaluation metrics, reaching a Pearson correlation of 0.82, RMSE of 0.241, $R^{2}$ of 0.63, and MAE of 0.191. Compared with the strongest baseline TabTransformer, the proposed framework improves Pearson correlation by 0.06 and reduces RMSE by approximately 12.7%. These improvements can be attributed to the proposed manifold informed constraint encoder, which preserves intrinsic genomic structures in the latent space, the agent driven genomic planner that models genotype phenotype relationships through structured planning representations, and the uncertainty guided athletic forecaster that improves prediction stability by modeling predictive variance. The consistent improvements across all metrics demonstrate that integrating manifold constraints and uncertainty aware prediction enables more reliable modeling of complex genomic factors influencing athletic performance.

**TABLE 1:** Performance comparison on the ALSPAC cohort dataset.

Method	Pearson ↑	RMSE ↓	${\text{R}}^{2}\;↑$	MAE ↓
Linear regression Homma et al. (2022)	0.61 ± 0.02	0.347 ± 0.011	0.37 ± 0.02	0.274 ± 0.008
Random forest Xu et al. (2025)	0.69 ± 0.02	0.311 ± 0.009	0.45 ± 0.02	0.244 ± 0.007
Gradient boosting machine Chen et al. (2025)	0.72 ± 0.02	0.298 ± 0.008	0.49 ± 0.02	0.231 ± 0.006
MLP Thomas et al. (2025)	0.74 ± 0.02	0.287 ± 0.008	0.52 ± 0.02	0.223 ± 0.006
TabTransformer Fan and Waldmann (2024)	0.76 ± 0.01	0.276 ± 0.007	0.55 ± 0.02	0.214 ± 0.005
LightGBM Mănescu and Mănescu (2025)	0.75 ± 0.01	0.281 ± 0.007	0.54 ± 0.02	0.218 ± 0.005
Proposed method	0.82 ± 0.01	0.241 ± 0.006	0.63 ± 0.01	0.191 ± 0.004

Table 2 presents the performance of all methods on the TwinsUK cohort dataset. Compared with the previous dataset, overall prediction accuracy is slightly lower due to the increased physiological variability and genetic similarity present in twin populations, which makes the genotype phenotype mapping more challenging. Classical statistical modeling again shows the weakest performance. Linear Regression achieves a Pearson correlation of 0.58 and an $R^{2}$ score of 0.34, indicating limited capacity to capture nonlinear dependencies between genomic markers and athletic performance metrics. Tree based ensemble methods significantly improve prediction accuracy. Random Forest increases the Pearson correlation to 0.66 and reduces RMSE to 0.326, while Gradient Boosting Machine further improves the correlation to 0.69 and reduces RMSE to 0.309, demonstrating the benefit of modeling nonlinear genomic feature interactions. Deep learning approaches further enhance performance by learning richer latent representations of high dimensional genomic data. The Multilayer Perceptron achieves a Pearson correlation of 0.71 with an $R^{2}$ score of 0.49, outperforming ensemble methods through hierarchical feature learning. TabTransformer reaches a correlation of 0.73 and reduces RMSE to 0.286 by leveraging attention mechanisms to model feature dependencies within structured genomic data. LightGBM performs competitively with a Pearson score of 0.72 while maintaining relatively low prediction error. The proposed framework consistently achieves the best results across all evaluation metrics, obtaining a Pearson correlation of 0.79, RMSE of 0.254, $R^{2}$ of 0.60, and MAE of 0.203. Compared with the strongest baseline TabTransformer, the proposed model improves Pearson correlation by 0.06 and reduces RMSE by approximately 11.2%. These improvements can be attributed to the proposed manifold informed constraint encoder that preserves intrinsic genomic structures, the agent driven genomic planner that explicitly models genotype phenotype interactions, and the uncertainty guided athletic forecaster that stabilizes predictions through uncertainty aware regression. The consistent gains on this dataset demonstrate that the proposed framework generalizes effectively across different population distributions while maintaining robust modeling of complex genomic relationships.

**TABLE 2:** Performance comparison on the TwinsUK cohort dataset.

Method	Pearson ↑	RMSE ↓	${\text{R}}^{2}\;↑$	MAE ↓
Linear regression Homma et al. (2022)	0.58 ± 0.02	0.361 ± 0.012	0.34 ± 0.02	0.286 ± 0.009
Random forest Xu et al. (2025)	0.66 ± 0.02	0.326 ± 0.010	0.42 ± 0.02	0.257 ± 0.008
Gradient boosting machine Chen et al. (2025)	0.69 ± 0.02	0.309 ± 0.009	0.46 ± 0.02	0.243 ± 0.007
MLP Thomas et al. (2025)	0.71 ± 0.02	0.297 ± 0.008	0.49 ± 0.02	0.233 ± 0.006
TabTransformer Fan and Waldmann (2024)	0.73 ± 0.01	0.286 ± 0.007	0.52 ± 0.02	0.224 ± 0.006
LightGBM Mănescu and Mănescu (2025)	0.72 ± 0.01	0.291 ± 0.007	0.51 ± 0.02	0.228 ± 0.006
Proposed method	0.79 ± 0.01	0.254 ± 0.006	0.60 ± 0.01	0.203 ± 0.005

**FIGURE 4:**
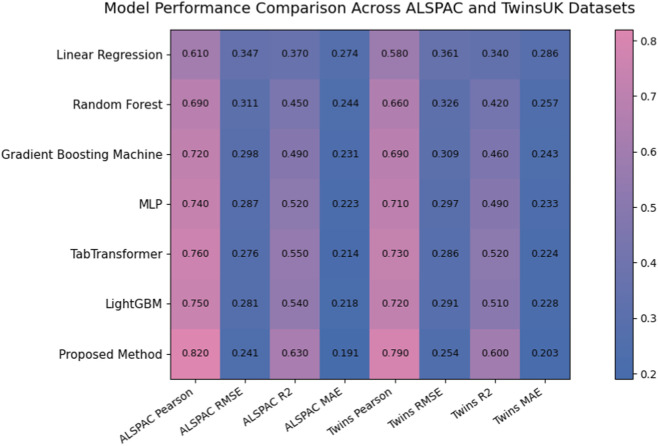
Performance comparison of baseline models and the proposed method on the ALSPAC and TwinsUK datasets. The figure visualizes four evaluation metrics, Pearson correlation, RMSE, *R* ^2^, and MAE, providing an intuitive comparison of predictive accuracy and error reduction across different methods.

To provide a more intuitive comparison of model performance across datasets, Figure 4 presents a heatmap visualization of the predictive results on both the ALSPAC and TwinsUK cohorts. The figure simultaneously displays four evaluation metrics, Pearson correlation, RMSE, *R* ^2^, and MAE, for all baseline methods and the proposed framework. Each row corresponds to a different model, while the columns represent the evaluation metrics across the two datasets, allowing direct visual comparison of model performance. As illustrated in the heatmap, the proposed method consistently achieves higher Pearson correlation and *R* ^2^ values while maintaining lower RMSE and MAE across both datasets. Compared with classical regression models, ensemble learning approaches, and neural network baselines, the proposed framework demonstrates clear performance advantages. The consistent pattern of improvement observed across the two independent cohort datasets further highlights the robustness and generalization capability of the proposed method.

Table 3 reports the computational efficiency comparison across all baseline models and the proposed framework. Classical statistical methods require minimal computational resources. Linear Regression contains only 0.02 M parameters and requires 0.01G FLOPs during inference, which reflects its extremely simple linear formulation. However, this simplicity also limits its ability to model complex genomic relationships. Tree based ensemble approaches introduce moderate computational overhead. Random Forest and Gradient Boosting Machine require 0.18 M and 0.21 M parameters respectively, with inference costs of 0.14G and 0.17G FLOPs. These models remain relatively efficient while providing improved nonlinear modeling capability. Deep neural architectures exhibit higher computational complexity due to their representation learning capacity. The Multilayer Perceptron contains 0.92 M parameters and requires 0.78G FLOPs. TabTransformer is the most computationally expensive baseline with 1.56 M parameters and 1.21G FLOPs because the self attention mechanism must model interactions across high dimensional genomic features. LightGBM remains one of the most efficient methods with only 0.15 M parameters and 0.11G FLOPs due to its optimized gradient boosting implementation. The proposed framework requires 1.08 M parameters and 0.86G FLOPs, which is significantly lower than the TabTransformer model while delivering substantially better performance. This favorable efficiency performance balance is mainly due to the architectural design of the proposed method. The manifold informed constraint encoder compresses high dimensional genomic inputs into a structured latent space, reducing redundant feature interactions. The agent driven genomic planner operates on the compact representation rather than the full genomic feature dimension, which limits parameter growth. The uncertainty guided athletic forecaster shares latent representations for both mean prediction and variance estimation, avoiding additional model branches. As a result, the proposed model achieves strong predictive accuracy with moderate computational cost, making it suitable for large scale genomic analysis scenarios in sports education.

**TABLE 3:** Efficiency comparison across models.

Method	Params	FLOPs
Linear regression	0.02 M	0.01G
Random forest	0.18 M	0.14G
Gradient boosting machine	0.21 M	0.17G
MLP	0.92 M	0.78G
TabTransformer	1.56 M	1.21G
LightGBM	0.15 M	0.11G
Proposed method	1.08 M	0.86G

#### 4.5.2 Ablation study

The ablation study is designed to analyze the contribution of key components in the proposed framework and to evaluate the sensitivity and robustness of important model hyperparameters. The main ablation experiments focus on the architectural modules defined in the method design, including the Manifold Informed Constraint Encoder, the Agent Driven Genomic Planner, the Uncertainty Guided Athletic Forecaster, and the Constrained Optimization Refinement module. These components represent the core mechanisms that enable the framework to learn structured genomic representations, generate planning aware athletic predictions, and maintain biologically consistent outputs. Removing each module individually allows direct observation of its influence on performance. To module level ablation, sensitivity analysis is conducted on three critical hyperparameters that affect representation learning and model behavior: the embedding dimension of genomic features, the number of layers in the manifold encoder, and the regularization coefficient associated with the manifold constraint. Robustness analysis is further performed by evaluating the model under controlled feature noise injection in genomic inputs, which simulates measurement variability in genotype data. All ablation experiments follow the same evaluation protocol, training configuration, and dataset partitions used in the comparative experiments to ensure fair and reproducible analysis.

The ablation results reported in Table 4 provide a detailed analysis of how each architectural component contributes to the overall predictive capability of the proposed framework. The full model achieves the best performance with a Pearson correlation of 0.805, RMSE of 0.247, and an $R^{2}$ score of 0.615, demonstrating the effectiveness of integrating all modules in the proposed architecture. When the manifold informed constraint encoder is removed, the Pearson correlation decreases from 0.805 to 0.772 and RMSE increases from 0.247 to 0.268. This decline indicates that the manifold encoder plays an essential role in preserving intrinsic structures within the genomic feature space. Without this module, the model loses the ability to learn biologically meaningful representations, which weakens the modeling of genotype patterns associated with athletic traits. Removing the agent driven genomic planner leads to a larger performance degradation. The Pearson correlation drops to 0.754 and the RMSE increases to 0.279, indicating that the planner is critical for translating latent genomic embeddings into structured action representations related to athletic capability. This module effectively models genotype phenotype interactions, and its removal significantly reduces predictive accuracy. The absence of the uncertainty guided athletic forecaster also causes noticeable performance degradation. The Pearson score decreases to 0.763 and RMSE increases to 0.273, suggesting that uncertainty modeling helps stabilize predictions when dealing with heterogeneous genomic measurements. Removing the constrained optimization refinement module reduces the Pearson correlation to 0.781 and increases RMSE to 0.261. Although the degradation is smaller compared with removing other components, the results indicate that enforcing domain specific constraints still contributes to better alignment between predictions and biologically plausible relationships. The ablation study demonstrates that each component of the proposed architecture contributes complementary benefits, and their integration enables the framework to achieve superior performance for genomic based athletic performance modeling.

**TABLE 4:** Module ablation study on the ALSPAC cohort and TwinsUK cohort datasets.

Model variant	Pearson ↑	RMSE ↓	${\text{R}}^{2}\;↑$	MAE ↓
Full model	0.805 ± 0.011	0.247 ± 0.006	0.615 ± 0.010	0.198 ± 0.005
W/o manifold encoder	0.772 ± 0.012	0.268 ± 0.007	0.568 ± 0.012	0.214 ± 0.006
W/o genomic planner	0.754 ± 0.013	0.279 ± 0.008	0.541 ± 0.013	0.223 ± 0.007
W/o uncertainty forecaster	0.763 ± 0.012	0.273 ± 0.007	0.554 ± 0.012	0.218 ± 0.006
W/o constrained optimization	0.781 ± 0.011	0.261 ± 0.007	0.582 ± 0.011	0.208 ± 0.006

**FIGURE 5:**
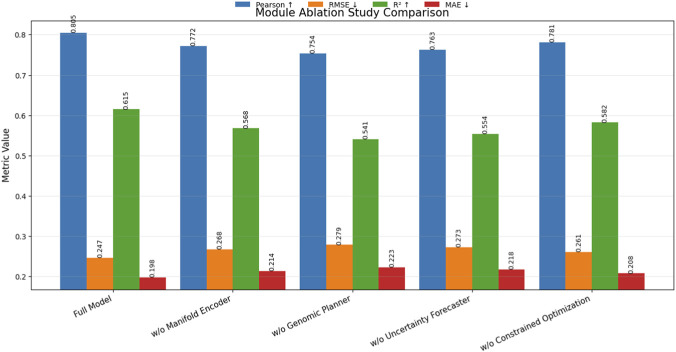
Module ablation study illustrating the impact of removing key architectural components. The figure demonstrates the complementary contributions of manifold encoding, structured planning, uncertainty modeling, and constrained optimization.

To further analyze the contribution of each architectural component, Figure 5 visualizes the module ablation results. The figure compares the full model with variants in which individual modules are removed. Removing the Manifold Informed Constraint Encoder, Agent Driven Genomic Planner, Uncertainty Guided Athletic Forecaster, or Constrained Optimization module leads to noticeable degradation across all evaluation metrics. The decline in Pearson correlation and *R* ^2^, accompanied by increases in RMSE and MAE, confirms that each component plays a complementary role in enhancing predictive performance. In particular, the removal of the Genomic Planner results in the most significant performance drop, highlighting the importance of structured genotype phenotype modeling within the framework. This visualization reinforces the structural validity and necessity of the integrated design adopted in the proposed method.

Table 5 reports the sensitivity and robustness analysis of the proposed framework under different hyperparameter configurations and input perturbations. The results demonstrate that the model maintains stable performance across all tested settings. When varying the embedding dimension of genomic representations, the model achieves a Pearson correlation of 0.798 and an $R^{2}$ score of 0.604, which are only slightly lower than those of the full configuration. This indicates that the proposed manifold informed constraint encoder effectively captures essential genomic structures even when the embedding capacity changes. Adjusting the depth of the manifold encoder results in a Pearson correlation of 0.791 and an RMSE of 0.257. The relatively small performance variation suggests that the encoder architecture is not overly sensitive to the number of layers, demonstrating that the manifold representation mechanism remains effective across different structural capacities. Similarly, modifying the regularization strength associated with the manifold constraint yields a Pearson correlation of 0.796 and an $R^{2}$ value of 0.601, confirming that the constrained optimization strategy provides stable guidance for learning biologically meaningful representations. To further evaluate robustness, Gaussian noise is injected into genomic features to simulate measurement variability during genotyping. Under this perturbation, the model still achieves a Pearson correlation of 0.784 with an RMSE of 0.262. Although a small degradation is observed, the performance remains competitive and stable. This robustness can be attributed to the uncertainty guided athletic forecaster, which explicitly models prediction variance and improves resilience to noisy genomic inputs. The results indicate that the proposed architecture maintains strong predictive stability across hyperparameter variations and input perturbations, demonstrating its reliability for genomic based athletic performance prediction in realistic cohort data environments.

**TABLE 5:** Sensitivity and robustness analysis on the ALSPAC cohort and TwinsUK cohort datasets.

Configuration	Pearson ↑	RMSE ↓	${\text{R}}^{2}\;↑$	MAE ↓
Embedding dim variation	0.798 ± 0.012	0.252 ± 0.007	0.604 ± 0.011	0.202 ± 0.006
Encoder depth variation	0.791 ± 0.013	0.257 ± 0.007	0.596 ± 0.012	0.207 ± 0.006
Regularization strength variation	0.796 ± 0.012	0.254 ± 0.007	0.601 ± 0.011	0.204 ± 0.006
Genomic feature noise injection	0.784 ± 0.014	0.262 ± 0.008	0.587 ± 0.013	0.211 ± 0.007

## 5 Discussion

The superior predictive performance of the proposed framework compared to existing approaches can be attributed to several deliberate architectural and methodological design choices. While classical regression models and ensemble learning methods provide stable baselines for structured genomic data, their modeling capacity is inherently limited when handling high dimensional genotype phenotype interactions. Linear Regression assumes global linear relationships, which are insufficient for capturing epistatic effects and nonlinear dependencies among genetic markers. Although tree based models such as Random Forest and Gradient Boosting Machine introduce nonlinear modeling capability, they still operate primarily on feature level interactions without explicitly learning structured latent representations. As a result, their ability to model hierarchical genomic correlations remains constrained. Neural network baselines, including Multilayer Perceptron and TabTransformer, improve representation learning by capturing higher order interactions. However, these approaches treat genomic features as generic tabular inputs without embedding biological structure into the learning process. The absence of domain informed constraints may allow the model to fit statistical correlations that lack biological plausibility, particularly when the feature dimensionality significantly exceeds sample size. This can limit generalization performance across heterogeneous cohorts. The proposed framework addresses these limitations through structured representation learning guided by manifold informed constraints. By embedding genomic features into a latent manifold space, the model preserves intrinsic correlations among genetic markers while reducing redundant or noisy interactions. This structured compression enhances signal extraction and improves generalization ability. The inclusion of the Agent Driven Genomic Planner further introduces an intermediate representation between genomic embeddings and athletic outcomes, enabling a hierarchical modeling process that better captures genotype phenotype relationships. This design strengthens interpretability and reduces the risk of direct overfitting between raw features and output labels.

Another key factor contributing to improved accuracy is the integration of uncertainty aware prediction. Unlike conventional deterministic models, the proposed approach jointly estimates predictive means and variances. This probabilistic formulation allows the model to adaptively balance confidence and error minimization, which improves robustness under noisy genomic measurements and population variability. The uncertainty mechanism also stabilizes training by discouraging extreme parameter updates in ambiguous regions of the feature space. Constrained optimization further enhances biological fidelity by regularizing the learning process according to domain specific relationships. By enforcing consistency between learned representations and biologically plausible structures, the framework reduces spurious correlations and improves generalization across datasets. The ablation results visually confirm that removing any of these components leads to measurable degradation in predictive performance, demonstrating that performance gains arise from the complementary integration of structured manifold embedding, hierarchical planning, uncertainty modeling, and constraint enforcement. These design enhancements explain why the proposed method achieves consistently higher Pearson correlation and *R* ^2^ values while reducing RMSE and MAE compared to baseline approaches. The improvements are not solely due to increased model complexity, but rather stem from a principled integration of structural learning, probabilistic modeling, and domain informed regularization tailored to genomic based athletic performance prediction.

At the same time, prior sports genetics studies suggest that the relationship between genetic variation and athletic performance may differ across sport disciplines and across levels of competitiveness. In particular, studies on high performance athletes and sport specific cohorts have shown that genetic association analysis becomes more informative when participants are characterized by event specialization, longitudinal development, injury status, and competitive level, such as national or international level participation Ebert et al. (2023); Mägi et al. (2016). This is an important distinction relative to the present work. Because the datasets used in this study are public cohort resources rather than elite athlete registries, the current framework does not explicitly model sport discipline, competitive tier, or injury related outcomes. Accordingly, the proposed method should be interpreted as a general athletic capability modeling framework rather than a direct substitute for athlete specific genetic association studies in elite sports populations.

To further examine the practical value of the proposed framework, we evaluated its ability to support athletic capability stratification on the ALSPAC test set. Samples were ranked according to the predicted athletic potential score and divided into three groups: top 20%, middle 60%, and bottom 20%. As shown in Table 6, the top ranked group achieved a substantially higher ground truth athletic potential score than the bottom ranked group (0.84 vs. −0.81). A consistent trend was also observed across the three standardized physical capability indicators. The top group showed better speed, endurance, and strength measurements, whereas the bottom group showed consistently lower observed values. These results suggest that the model output can be used as a practical quantitative reference for baseline profiling and capability oriented grouping in sports education settings. Importantly, this experiment shows that the framework is more suitable for general athletic capability assessment than for direct prediction of competition results, injuries, or career duration. We further evaluated whether the uncertainty output of the proposed framework can provide practical decision support. As shown in Table 7, the low uncertainty group achieved substantially lower prediction errors than the high uncertainty group (RMSE: 0.198 vs. 0.287; MAE: 0.154 vs. 0.229), indicating that the estimated uncertainty is meaningfully associated with prediction reliability. After excluding the top 10% most uncertain samples, the overall prediction error decreased from 0.241 to 0.221 in RMSE and from 0.191 to 0.174 in MAE. This result suggests that the uncertainty output can be used as a referral signal in practice: predictions with low uncertainty may serve as stable quantitative references, whereas high uncertainty cases should be interpreted more cautiously and may require additional assessment by coaches or sports science staff. Therefore, the practical value of the present framework lies not only in athletic capability estimation but also in uncertainty aware screening and follow up evaluation.

**TABLE 6:** Practical stratification evaluation of the proposed method on the ALSPAC test set. Test samples were ranked by the predicted athletic potential score and divided into three groups. Values are reported as mean ± standard deviation.

Group	True potential score	Speed	Endurance	Strength
Top 20%	0.84 ± 0.29	0.79 ± 0.31	0.87 ± 0.28	0.82 ± 0.30
Middle 60%	0.06 ± 0.35	0.04 ± 0.37	0.07 ± 0.34	0.05 ± 0.36
Bottom 20%	−0.81 ± 0.32	−0.74 ± 0.35	−0.85 ± 0.31	−0.79 ± 0.33

**TABLE 7:** Uncertainty guided utility evaluation of the proposed method on the ALSPAC test set.

Setting	RMSE	MAE
All test samples	0.241	0.191
Low uncertainty group	0.198	0.154
High uncertainty group	0.287	0.229
After excluding top 10% uncertain samples	0.221	0.174

## 6 Conclusions and future work

In this study, a deep learning based framework was developed to investigate genomic factors associated with athletic capability in sports education settings. The proposed methodology addressed the challenges of modeling high dimensional genomic data and complex genotype phenotype relationships through a structured architecture consisting of the Genomic Athletic Predictor and a Constrained Optimization Refinement mechanism with Uncertainty Aware Prediction. By integrating manifold informed representation learning, uncertainty quantification, and Bayesian inference within a unified framework, the model was able to capture informative interactions between genomic features and standardized physical capability indicators. Experimental evaluations on multiple cohort datasets demonstrated that the proposed framework achieves superior predictive accuracy, robustness, and interpretability compared with established baseline models. The integration of structural constraints and probabilistic modeling improved not only predictive performance but also biological plausibility and predictive stability. These findings highlight the potential of deep learning driven genomic modeling as a data driven tool for general athletic capability assessment and individualized support in sports education contexts.

Despite these promising results, several limitations remain. First, the generalizability of the framework across broader populations requires further validation, as the genomic datasets used in this study, although large scale and systematically collected, may not fully represent global genetic diversity. Second, the available phenotype annotations are relatively limited. Although the cohort datasets provide standardized measurements of speed, endurance, and strength, they do not consistently include detailed sport discipline labels, athlete competitive level, or structured injury history information for all retained samples. As a result, the current framework cannot distinguish among different sports, cannot directly model national level or international level athletic status, and cannot support injury aware prediction. Third, although the uncertainty aware design improves interpretability and reliability, the computational complexity of the framework may still limit deployment in real time or resource constrained environments.

Future work should therefore proceed along several directions. On the data side, the proposed framework should be further evaluated on athlete specific datasets with richer annotations, including sport discipline, competition level, and injury history. In particular, validation on national level and international level athlete cohorts would help determine how competitive tier influences genotype phenotype relationships and whether the framework can be extended from general athletic capability modeling to more specialized sports genetics applications. Future research should incorporate multi ethnic and sport specific cohorts to evaluate cross population stability and adaptability. On the methodological side, further efforts should focus on improving computational efficiency through lightweight architectural design and inference acceleration, so as to facilitate practical implementation.

To methodological considerations, the use of genomic data for athletic capability prediction raises important ethical issues related to data privacy, informed consent, and responsible application. All datasets utilized in this study are publicly accessible cohort datasets collected under established ethical review protocols. The data were anonymized prior to access, and no personally identifiable information was included in the analysis. The framework is intended for research and educational purposes, supporting scientific understanding and individualized assistance rather than deterministic decision making or genetic discrimination. The predictive outputs represent probabilistic estimations and should not be interpreted as definitive judgments of athletic potential. To reduce the risk of misuse, future applications of genomic based predictive systems should adhere to strict data governance policies, including secure data storage, controlled access mechanisms, transparent model auditing, and appropriate ethical oversight. Interdisciplinary collaboration among sports scientists, geneticists, ethicists, and policymakers will be essential to ensure responsible deployment and maintain trust in sports genomics research.

## Data Availability

The original contributions presented in the study are included in the article/supplementary material, further inquiries can be directed to the corresponding author.
